# Effect of High Molecular Weight PPTA on Liquid Crystalline Phase and Spinning Process of Aramid Fibers

**DOI:** 10.3390/polym12051206

**Published:** 2020-05-25

**Authors:** Cuiqing Teng, Hui Li, Jing Liu, Hao Gu, Haijuan Kong, Muhuo Yu

**Affiliations:** State Key Laboratory for Modification of Chemical Fibers and Polymer Materials, College of Materials Science and Engineering, Donghua University, Shanghai 201620, China; huili0512@163.com (H.L.); ljingdoris@163.com (J.L.); guhao91622@163.com (H.G.); khj3155@126.com (H.K.); yumuhuo@dhu.edu.cn (M.Y.)

**Keywords:** PPTA, liquid crystalline, fiber, mechanical property

## Abstract

High molecular weight poly (*p*-phenylene-terephthalamide) (h-PPTA) was blended with the commercial PPTA in concentrated sulfuric acid to improve the spinnability of the polymer solutions and the mechanical properties of the as-spun fibers. h-PPTA in the solution has an influence on the temperature of the formation of liquid crystalline phenomenon. The temperature range with the existence of the liquid crystalline phase increases upon the contents of h-PPTA in the solution, and the extended temperature window is helpful for the preparation of PPTA fibers by the dry-jet wet-spinning technology. The long-chains of h-PPTA enhance the inter-macromolecular interactions and induce the orientation of short-chains for PPTA along the fiber axis under the shear stress in the spinneret and the stretching stress at the air gap. These effects also increase the maximum drawing ratio in the spinning process and improve the mechanical properties of the obtained fibers. The crystallinity and crystal orientation of the fibers are investigated by X-ray diffraction, and results from sonic velocity test further confirm ordering state of the macromolecular chains. The fibril morphologies of the fibers are also studied by a scanning electric microscope.

## 1. Introduction

Developing stronger high-performance materials with a lighter weight are of significance for broadening their applications in many areas. Among them, high-performance polymer fibers have attracted great attention due to their high specific strength (the ratio of tensile strength to density) and the ability to withstand high impact. These high-performance fibers, such as poly (*p*-phenylene-terephthalamide) (PPTA) fiber, poly-*p*-phenylene benzobisoxazole (PBO) fiber, ultrahigh molecular weight polyethylene (UHMWPE) fiber, etc., have been extensively used in numerous fields including aerospace, automotive, civil engineering, sport equipment and advanced composites. PPTA fiber, earlier developed by the Dupont in 1972 (Kevlar^®^) [1], is particularly known as the representative of the high-strength and high-modulus fibers. The specific tensile strength of Kevlar^®^ is 5 times than that of steel, which could be attributed to its high macromolecular orientations and strong inter-chain interactions including hydrogen bonding and aromatic stacking. The closely packed rod-like molecular chains and their stronger interactions could result in the liquid crystalline characteristics of the polymer solution, which has significant influences on the fiber processing and the mechanical properties of prepared fibers [2,3,4].

Many researches focused on the further enhancement of the mechanical properties of PPTA fibers, including the tensile strength and modulus [5,6,7,8]. Introducing the nanofiller on the surface or internal of the fibers is the one of most commonly strategies, which is expected to reinforce the aramid fiber by combining the two strong materials. O’Connor et al. [9] reported the physical coating of a carbon nanotube (CNT) onto the surface of Kevlar by swelling the fiber in the suspensions of CNT, demonstrating the ability of this novel additive for improving the mechanical properties of the fibers. However, CNT has to be prepared or modified in concentrated sulfuric acid at high temperature (100 °C), and PPTA chains will be inevitably degraded under this condition. Reinforcing organic fibers by incorporating the nanofillers in the spinning solution also have been reported. The tensile strength of the PBO/SWNT fiber containing 10 wt % single-wall carbon nanotubes (SWNTs) is about 50% higher than that of the PBO fibers without SWNTs [10]. The ultimate tensile strength of the drawn UHMWPE fibers containing functionalized nanosilica reaches 7.6 GPa, which is about 2.3 times of that of the parallel fiber without the addition of any nanofillers [11]. Modified graphene oxide (GO) is also reported to be incorporated in the poly(vinyl alcohol) (PVA) to prepare PVA composite fibers, and the tensile strength and initial modulus of these drawn fibers with the GO loading of 0.60 wt % increase by 39% and 69%, respectively, compared with those of neat drawn PVA fiber [12]. However, the incorporation of these rigid nanofillers hampers the orientation of macromolecular chains in the stretching process, and even results in the formation of defects and stress concentrations since there exists a stress transfer between the polymer chains and nanofillers. Moreover, the dispersion of the nanofillers in polymer solutions with high viscosity is still one of the key challenges in industrial applications. Therefore, this blending method to enhance the properties of high-performance fibers with the introduction of inorganic nanofillers is unavailable for large scale productions.

Mechanical properties of organic fibers are greatly dependent on the molecular weight and its distribution of the polymers, and high molecular weight polymers are usually used to produce strong fibers. The viscosity of the polymer solution is mainly determined by the molecular weight of polymers, which has an obvious influence on the spinning process. Therefore, the molecular weight could not to be unlimitedly increased due to the extremely high viscosity, which is unavailable for the spinning process to prepare fibers. Alternatively, it is an effective approach to enhance the mechanical properties of the fibers by incorporating a high molecular weight polymer in the spinning solutions with the consideration of the spinnability and the strength. As reported by Xu et al. [13], the mixture of UHMWPE with high density polyethylene (HDPE) could modify the flowability of pristine HDPE and then reinforce the obtained fibers. Aguilar et al. [14] blended a small amount of UHMWPE with the metallocene polyethylene, and the spinnability of the blended melt and the surface morphologies of the extruded filament were gradually improved with the increased contents of UHMWPE. Pan et al. [15,16] added ultrahigh molecular weight polyacrylonitrile (UHMW-PAN) into the spinning solution of PAN in dimethyl sulfoxide, and the steady-state and dynamic rheological behaviors of the blended system were investigated in detail. As a result, the incorporation of an appropriate UHMW-PAN was beneficial to reduce the ratio of the surface tension (α) between the flowed filament and coagulation bath to the viscosity (*η*), demonstrating the improved spinnability and enhanced stability of the blended polymer solutions. Auhl et al. [17] studied the nonlinear rheological responses of molten binary blends of polymers with long and short chains, and predicted a novel mechanism whereby the effective stretch relaxation time of the long chains in the bimodal blend could be enhanced substantially, which is higher than its value in the pure melt.

In this report, we added high molecular weight PPTA (h-PPTA) in the industrial PPTA to prepare spinning dope in concentrated sulfuric acid, in which [*η*]s of PPTA and h-PPTA is 5.8 dL/g and 8.9 dL/g, respectively. An appropriate addition of h-PPTA (<10 wt %), to a certain extent, has an influence on the rheological behaviors and spinnability of the polymer solutions, and the introduction of h-PPTA could significantly enhance the orientation of molecular chains and mechanical properties of the prepared fibers. As far as we know, this strategy of blending with h-PPTA and PPTA to prepare the fibers with enhanced mechanical properties has not been reported yet. It is of importance to understand the changes of the liquid crystalline behaviors, spinning parameters, chain orientations and crystallinity structures upon the increasing content of h-PPTA in these blended systems.

## 2. Materials and Methods

### 2.1. Materials

PPTA powder with the intrinsic viscosity ([*η*]) of 5.8 dL/g and high molecular weight PPTA (h-PPTA) with [*η*] of 8.9 dL/g were kindly supplied by Hebei Silicon Valley Chemical Co. Ltd., Handan, China. The powders of two resins were dried in a vacuum oven at 100 °C for 8 h to remove the residue water prior to use. Concentrated sulfuric acid (H_2_SO_4_, 98%) and fuming H_2_SO_4_ (120%) were purchased from Jiangsu Kunshan Jingke Microelectronic Materials Co. Ltd., Suzhou, China.

### 2.2. The Dissolution of PPTA and h-PPTA/PPTA

The solvent of H_2_SO_4_ with the concentration of 99.5%–100% to dissolve the polymers could be obtained by blending the concentrated H_2_SO_4_ and fuming H_2_SO_4_ with a certain proportion. PPTA was firstly added in the above solvent at low temperature, and then transferred to a flask for further dissolution at 90 °C. In this dissolution process, PPTA resin in the mixed H_2_SO_4_ was initially swelled with the mixture changing to a slurry state within 5 min. Then the PPTA particles gradually reduced and completely dissolved after 1 h, exhibiting a liquid crystalline phenomenon. The blended solutions of h-PPTA/PPTA were prepared using the similar method, and the mass fractions of h-PPTA in the polymers were 3 wt %, 5 wt %, 8 wt % and 10 wt %, respectively, which could also be observed with the liquid crystalline phenomenon.

### 2.3. The Spinning Process of Fibers

**Figure 1:**
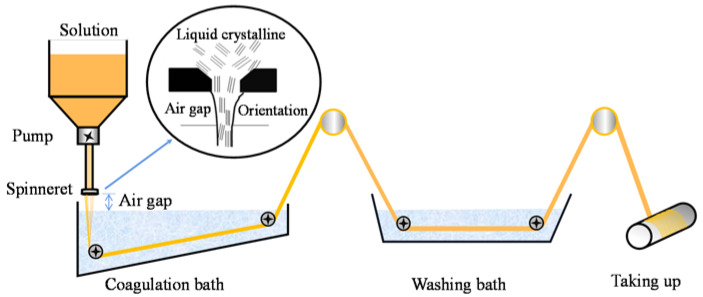
Schematic diagram of the dry-jet wet-spinning process.

The PPTA and h-PPTA/PPTA fibers were fabricated through a dry-jet wet-spinning process as illustrated in Figure 1. h-PPTA/PPTA/H_2_SO_4_ mixtures with various ratios in a slurry state were firstly put into a twin screw at 90 °C to obtain the uniform spinning dopes. The dopes were filtrated and used to prepare the fibers on a spinning machine, which was designed in our laboratory. As shown in the schematic diagram (Figure 1), the dopes were extruded through a spinneret with an air gap and entered into the coagulation bath with the following washing and drying process to obtain the aimed fibers. The spinning parameters were listed as follows: the diameter of the spinneret is 0.15 mm, the drawing ratio is 2:1, the temperature of twin screw to dissolve the mixture is 90 °C, the air gap is 10 mm, the coagulation bath is dilute H_2_SO_4_ solution of 10 wt % and the temperature is 5–7 °C. The obtained fibers were then thermally treated with a fixed stress of 0.2 cN/dtex under nitrogen atmosphere at 500 °C for 10 s.

### 2.4. Characterization

The molecular weight (*M*_w_) and polydispersity index (PDI) were measured by *n*-alkylated PPTA using gel permeation chromatography (GPC, Waters, Milford, MA, USA) [18]. Then the measured *M*_w_ of PPTA and h-PPTA were 36,500 and 60,300, respectively, and the corresponding PDIs were 1.92 and 2.03, respectively. Polarizing optical microscope (POM) of Olympus BX 51-P was employed to observe the liquid crystalline phenomena of the PPTA/H_2_SO_4_ and h-PPTA/PPTA/H_2_SO_4_ solutions with various components. A small amount of the solution was dipped on a slide glass and shear stress was applied on the solution by sliding the covered glass to induce a certain orientation of the molecular chains. Then the sample was put on the hot stage of POM equipped by the Linkam temperature-controlled system. Colorful stripes would be observed by adjusting the temperature and the position of sample. The wide angle X-ray diffraction measurement was carried out using a Rigaku D/max-2550PC X-ray diffractometer system with CuK_α_ radiation and its wavelength of 0.15406 nm. The morphology of prepared fibers was observed by scanning electron microscope (SEM; Hitachi SU8010, Tokyo, Japan). The intrinsic viscosity [*η*] was obtained by extrapolating the reduced viscosity measured in a capillary viscosimeter to zero concentration. The mechanical properties of the fibers were measured with a tensile testing machine of A0-3000cN with a drawing rate of 50 mm/min and a gauge length of 100 mm, and the average value was obtained by 20 times tests for each sample. To further evaluate the orientation state of the fibers, sonic velocity along the fiber’s axial was measured at 25 °C under the relative humidity of 60% on a SCY-III sonic orientation instrument designed in our laboratory with a frequency of 10 KHz. The fiber with 80 cm long was put on the instrument to test the propagation time of sound wave at the fiber position of 20 cm and 40 cm, respectively, and the orientation factor can be calculated using Equation (5) in Section 3.3.

## 3. Results and Discussion

### 3.1. Liquid Crystalline Phenomenon

The rigid-rod characteristics of the PPTA chemical structures and the strong intermolecular interactions of their molecular chains result in a very high melting point of the polymer, even beyond the decomposition temperature, which indicates that the aramid fibers could not be produced by the commercially used melt-spinning process. In comparison, solvent-based spinning without excessively high temperature is an alternative route to fabricate such high-performance fibers [19]. Even though concentrated sulfuric acid is highly corrosive for all of the parts in the spinning line, such as storage tanks, tubes, pumps, filters, spinnerets, washing bathes, etc., the solvent is relatively nontoxic, non-flammable and non-volatile and has been produced on a large scale. The dependence of the viscosity on the concentration for PPTA solution in H_2_SO_4_ could be divided into two domains. One is at the concentration region lower than 10 wt % in which the apparent viscosity increases with the increase in content of the solution, and the solution in this region shows the isotropic features, i.e., the PPTA molecules have no long-range orientational order in such state. The other domain is at the concentration of 13–20 wt % for the polymer solution, which displays reduced apparent viscosity due to the formation of liquid crystalline nematic state in the solution, indicating the appearance of molecular orientational order of PPTA. On the other hand, the liquid crystalline state is also affected by the temperature and molecular weight of the polymer. From our previous work and experiences, 18.5 wt % of the polymer concentration is selected to investigate the solution properties and spinnability in the following discussion.

**Figure 2:**
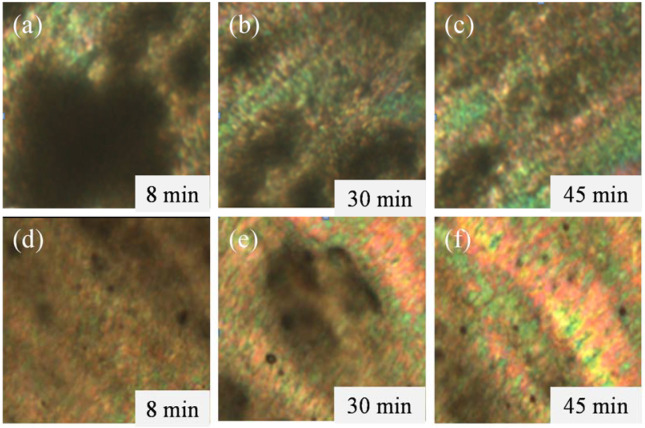
Polarizing optical microscope (POM) images of the solutions at various dissolved time. (**a**–**c**) PPTA/H_2_SO_4_ solution with dissolved time of 8, 30 and 45 min respectively, and (**d**–**f**) h-PPTA/PPTA-H_2_SO_4_ solution with 8 wt % h-PPTA in the polymer.

Figure 2a–c displays the POM images of PPTA dissolved in H_2_SO_4_ for 8, 30 and 45 min, with a concentration of 18.5 wt % at 90 °C. It is clearly shown the colorful stripes on the polarizing microscope, indicating the formation of liquid crystalline phase in PPTA/H_2_SO_4_ solution with a certain orientation in the direction of sliding the cover glass under a shear stress. The solution of the h-PPTA/PPTA/H_2_SO_4_ containing 8 wt % h-PPTA shows a similar liquid crystalline behavior, as shown in Figure 2d–f. Although many incompletely dissolved polymers with the dissolution time of 8 minutes could be noticed in Figure 2d, the liquid crystal phenomenon also could be observed. With the extension of the dissolution time for h-PPTA/PPTA/H_2_SO_4_ system, the liquid crystal structure formed in the solution gradually improves, and the colorful stripe pattern in the POM images becomes more obvious. Compared to the PPTA/H_2_SO_4_ solution at the same dissolution, the h-PPTA/PPTA/H_2_SO_4_ solution seems to form more of a perfect liquid crystalline phase since the latter displays more colorful stripes.

**Figure 3:**
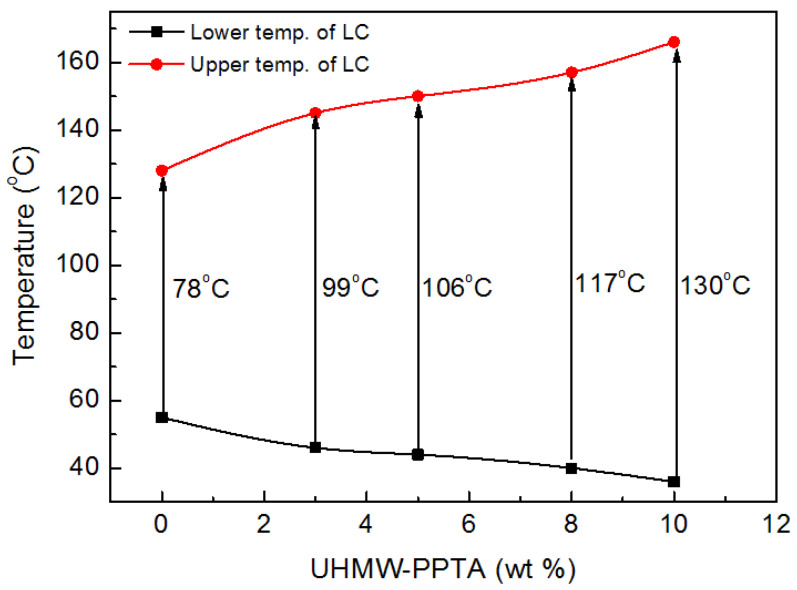
Temperature ranges of the formation of liquid crystalline phase for the solutions with various contents of h-PPTA.

The addition of h-PPTA has a significant influence on the temperature range of the formation of liquid crystalline phenomenon and the solutions are in the isotropic state outside the appropriate temperature region, i.e., the molecules have no long-range orientational order. Figure 3 summarizes the effect of various h-PPTA contents on the temperature range of the appearance for the liquid crystalline phase in the blend solutions with the solution concentration of 18.5 wt %. Apparently, with increasing content of h-PPTA in the solution, the lower temperature limits at which the liquid crystalline phenomenon appears gradually decrease and the higher temperature limits at which the liquid crystalline phase disappears progressively increase. In detail, the temperature range with the existence of liquid crystalline phase in the solution is about 78 °C for PPTA/H_2_SO_4_, while the value increases to 99 °C and 117 °C for h-PPTA/PPTA/H_2_SO_4_ solution with the content of 3 wt % and 8 wt % for h-PPTA, respectively. The reasons for this phenomenon could be attributed to two aspects. One is that the addition of higher molecular weight polymers with such rigid-rod like structures as PPTA means a large axial ratio and an obvious rigidity of the molecular chains, which is more conducive to form a completely straight rod-like configuration, thus resulting in the increased anisotropic volumes. On the other hand, the shear stress and stretching stress not only could induce the orientation of short molecular chains of pristine PPTA, but also promote their extension of the rod-shaped conformation with the “*Oriented Bridging*” of the long-chains for h-PPTA due to the strong intermolecular interactions between them. The widened temperature region for the formation of liquid crystalline phase in solution, i.e., the temperature range from the lower value limit for the appearance of liquid crystalline phase to the higher value limit for the disappearance of this phenomenon, is of significance for the processing of dry-jet wet-spinning of the high-performance fibers.

**Figure 4:**
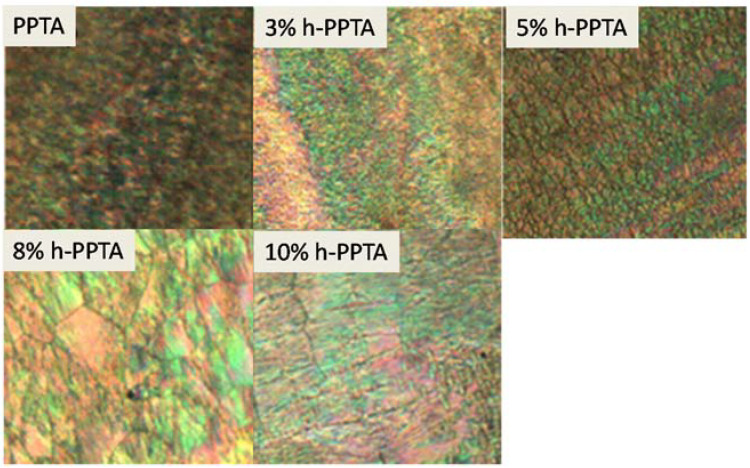
POM images of the blended solutions containing various contents of h-PPTA.

Figure 4 shows the liquid crystalline phenomenon of the blended solution of h-PPTA/PPTA/H_2_SO_4_ with various contents of h-PPTA at 90 °C. With the addition of the high molecular weight polymer of h-PPTA, the POM images show more colorful stripes, indicating the appearance of more prefect liquid crystalline phase of the blended solutions. Moreover, the sample containing 8 wt % fraction of h-PPTA exhibited “beautiful” stripes and relatively perfect liquid crystalline structures. As mentioned above, the addition of h-PPTA induces the orientation of the short molecular chains of PPTA along the direction of the applied stress and promotes the extension of the rod-shaped conformations. The diminished colorful strips with weakened liquid crystalline phenomenon for the solution with 10 wt % h-PPTA might be attributed to the influence of the solubility due to the increased concentration of polymers in solution.

### 3.2. Shear-Rate Dependent Viscosity

**Figure 5:**
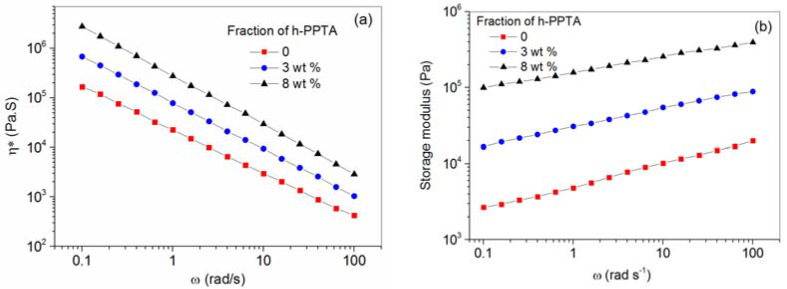
Complex viscosity (**a**) and storage modulus (**b**) as a function of frequency for the PPTA/H_2_SO_4_ solution and the blended h-PPTA/PPTA/H_2_SO_4_ solutions with 3 wt % and 8 wt % of h-PPTA.

The dependence of the viscosities on the shear-rate for the PPTA/H_2_SO_4_ solution and h-PPTA/PPTA/H_2_SO_4_ solutions with 3% and 8% contents of h-PPTA was further investigated. Figure 5 displays the complex viscosity (*η* *) and the storage modulus as a function of frequency for the above solution. Apparently, all the solutions exhibit the shear thinning behaviors, the addition of h-PPTA results in an increase of *η* * of the blended solutions at a fixed frequency. Similarly, as shown in Figure 5b, the storage modulus also increased upon the accelerated frequencies and the added contents of the high molecular weight polymer.

Relaxation time (*τ*) can be evaluated by the Cross-Williamson equation (Equation (1)) [20] as follows:

(1) $$\eta *=\frac{\eta_{0}}{1+{\left(\tau \;\omega \right)}^{1-m}}$$

where, *η*_0_ is the zero-shear viscosity, and *m* is the dimensionless parameter. As a result, the value of *τ* for PPTA/H_2_SO_4_ solution at 90 °C is 77 s, while the values of the blended solutions with 3 wt % and 8 wt % of h-PPTA increase to 89 s and 102 s, respectively. A relatively extended relaxation time of PPTA/H_2_SO_4_ solution indicates that the oriented PPTA chains need a longer time to release their orientations. Consequently, in the following spinning process of related fibers, the as-spun filaments should be solidified in an appropriate time duration, which should be much less than that of the relaxation time for polymer chains in order to hold their high orientations [21].

### 3.3. Fiber Preparation

**Figure 6:**
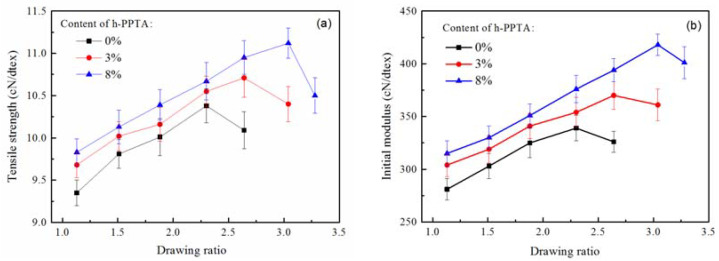
Effects of the drawing ratio at the air gap on the tensile strength (**a**) and the initial modulus (**b**) of the fibers.

During the dry-jet wet-spinning process, the fluid filament is injected through the spinneret with a distance of air gap before entering into the coagulation bath, in which the filament could not be solidified with the motionable features for the molecules and chain segments. The subsequent drawing process will cause the orientations of the molecular chains, leading to the improvement of tensile strength and modulus [22]. This drawn effect mainly occurs at the air gap in which the filament is in fluid state instead of solid conditions. As shown in Figure 6a, the maximum drawing ratio of PPTA fiber was 2.3 times with a tensile strength of 10.4 cN/dtex; whereas the maximum drawing ratio of the fibers containing 3 wt % and 8 wt % h-PPTA were 2.6 and 3.1 times, respectively, with the corresponded tensile strength of 10.9 and 11.5 cN/dtex, respectively. As shown in Figure 6b, the effects of h-PPTA on the initial modulus of the fibers exhibited a similar trend, demonstrating an increased maximum drawing ratio and the enhanced mechanical properties. Moreover, at a fixed drawing ratio, the addition of high molecular weight PPTA improved the tensile strength and initial modulus. As mentioned above, long-chains of h-PPTA molecules strengthened the interactions among the molecules and segments, which could endure a larger drawing ratio in the spinning process. Therefore, the addition of high molecular weight polymers can improve the spinnability of the liquid crystalline solution, and thus positively contribute to the strength of prepared fibers. 

**Figure 7:**
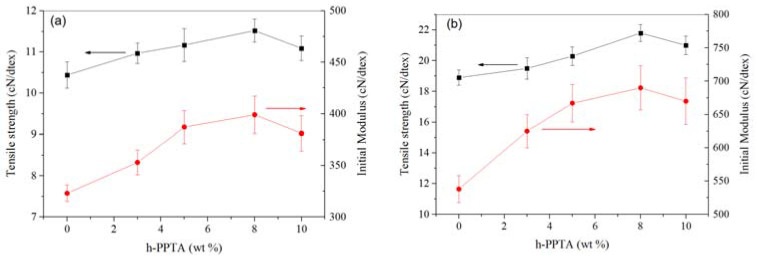
Plots of mechanical properties of as-spun fibers (**a**) and thermally treated fibers (**b**) vs. mass fraction of h-PPTA.

Figure 7 shows the effects of h-PPTA contents on the mechanical properties of the fibers. Apparently, both the tensile strength and initial modulus are enhanced upon increasing the contents of h-PPTA up to 8 wt %. As discussed above, the long-chains of h-PPTA exhibited the rod-like structures in the liquid crystalline spinning dope, and their strong intermolecular interactions induced the shorter PPTA chains to orderly arrange along the direction of the fiber axis under the shear stress in the spinneret and the stretching stress at the air gap. Meanwhile, the increased maximum drawing ratio is also favorable to enhance the mechanical properties of the fibers. However, the excessive addition of the high molecular weight polymer results in the significant increase of the viscosity for the spinning solutions, which definitely affect the fluidity of the spinning dope and the uniformity of the polymer solutions. When the content of h-PPTA was up to 10 wt %, the spinnability of the solution gets poor and the mechanical properties of obtained fibers declined. It is worth noting that the heat treatment of the fibers is one of the effective methods to prepare the high-modulus aramid fibers, such as PBO, Kevlar, Vactron, etc. In this work, the incorporation of high molecular weight polymer could enhance the modulus of the prepared fibers, which provided a new strategy for the preparation of the high-modulus fibers. Therefore, the as-spun aramid fiber was further treated at 500 °C, and the plot of mechanical properties vs. h-PPTA contents is illustrated in Figure 7b. Obviously, the mechanical properties of thermally treated fibers exhibited a similar changing trend on the h-PPTA contents. In detail, the tensile strength of thermally treated PPTA fiber was 18.9 cN/dtex, and the value increased to 21.8 cN/dtex for the fiber containing 8 wt % h-PPTA. Meanwhile, the initial modulus had a similar increase trend upon the h-PPTA content, and the value was up to 695 cN/dtex for the sample with 8 wt % h-PPTA. The mechanical properties of as-prepared fibers via our strategies were comparable or even higher than some commercially used aramid fibers, such as Kevlar^®^29 and Kevlar^®^49, i.e., the tensile strength and modulus of Kevlar^®^29 were 20.1 cN/dtex and 500 cN/dtex, respectively, and the values of Kevlar^®^49 were 19.3 cN/dtex and 850 cN/dtex, respectively.

### 3.4. Crystallinity and Orientation of the Fibers

**Figure 8:**
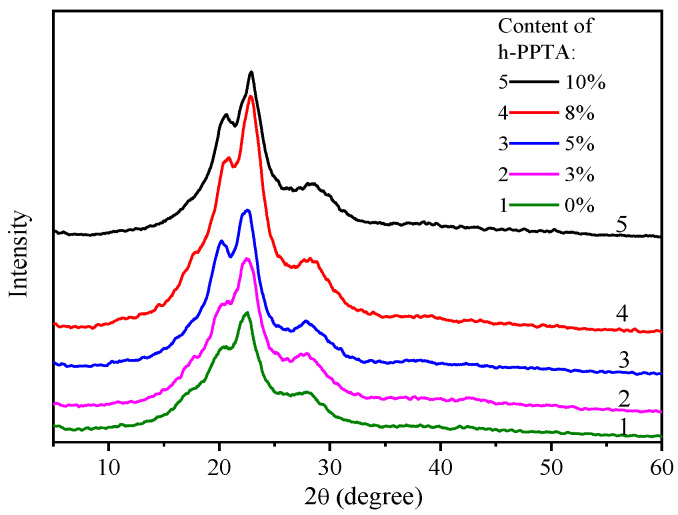
XRD spectra of the fibers with various content of h-PPTA.

The crystallinity is one of the important factors that will greatly affect the mechanical properties of the fibers. Figure 8 shows the XRD spectra of the fibers containing various fractions of h-PPTA. The strong diffraction peaks at 20.5°, 23.5° and 28.6° could correspond to the lattice planes of [110], [200] and [211], respectively [23]. The increase of h-PPTA contents in the fibers results in the sharper diffraction peaks, indicating the formation of the relatively perfect crystals [23]. Based on the three diffraction peaks with corresponded three lattice planes, the wide angle X-ray diffraction (WAXD) curves could be fitted with these peaks, and the crystallinity (*X*_c_) can be obtained by calculating the ratio of the crystalline-region to the whole area [24,25]. The calculated crystallinity values by the peak-fitting method are listed in Table 1. Apparently, the crystallinity exhibited an increasing trend upon the addition of h-PPTA. The incorporation of h-PPTA reduced the defects caused by the terminals of macromolecular chains, resulting in the increased crystallinity. Moreover, the long molecular chains of h-PPTA could promote the orientation degree of the shorter molecular chains for pristine PPTA at the shear stress in the capillary pores of spinnerets and the stretching stress in the air gap, inducing the formation of relatively perfect crystalline structures.

In order to quantitatively evaluate the crystal orientation along the fiber axis, the Hermans’ orientation factor (*f_c_*) was calculated from the [200] plane at 2*θ* = 23.5°, since this diffraction of the [200] plane is very noticeable and could be clearly isolated [25,26].

(2) $$f_{c}\cdot 100\%=[3<\cos^{2}\phi_{c}>-1]/2$$

where *f_c_* is the orientation factor along the fiber direction, *φ_c_* is the angle between the fiber axis and the *c* axis of crystal unit cell. The value of the mean-square cosines in this equation can be determined from the fully corrected intensity distribution reflected from this preferred crystalline plane, *I_c_* (*φ_c_*), averaged over the entire surface of the orientation sphere:

(3) $$<\cos^{2}\phi_{c}>=\frac{\int_{0}^{\frac{\pi }{2}}I(\phi_{c})\cos^{2}\phi_{c}\sin\phi_{c}d\phi_{c}}{\int_{0}^{\frac{\pi }{2}}I(\phi_{c})\sin\phi_{c}d\phi_{c}}$$

According to Equations (2) and (3), the calculated *f_c_* values of the PPTA fiber and h-PPTA/PPTA fibers are listed in Table 1. Apparently, the addition of h-PPTA in the fiber increased the crystal orientation. The orientation factor of the fiber containing 8 wt % h-PPTA in the [200] plane was 0.859, while the value of the PPTA fiber was only 0.804.

Sonic velocity along the fiber’s axial can also be used to evaluate the degree of orientation for the macromolecular chains. In the case of partially oriented polymer molecules, the molecular motion due to sound transmission has an angle direction along and across the molecular axis. The magnitude of either the along or across direction was taken to be a function of the angle between the molecular axis and the direction of sound propagation (*θ*). Then, there exists an expression for the sonic velocity and the *θ* (Equation (4)), and orientation factor (*f_s_*) of the sonic velocity can be calculated as the following equation (Equation (5)). [27]

(4) $$\cos^{2}\theta =1-\frac{2}{3}\frac{C_{u}^{2}}{C^{2}}$$

(5) $$f_{s}=1-\frac{C_{u}^{2}}{C^{2}}$$

where, *C* (km/s) is the sonic velocity along the fiber’s axis, and *C_u_* (km/s) is defined as the sonic velocity of the unoriented fibers, i.e., random orientational sample. Here, the sonic velocity of unoriented PPTA fiber was 1.57 km/s [28]. Therefore, the *f_s_* was easily calculated, which has been displayed in Table 1. The *f_s_* increased from 0.896 for the PPTA fiber without h-PPTA to 0.951 for the sample containing 8 wt % h-PPTA, which then decreased to 0.939 as continually raising the h-PPTA content to 10 wt %. The differences in the orientation factor along the fiber axis were more obvious for these samples compared to the result along the [200] plane. Therefore, this result further confirmed that the addition of h-PPTA was favorable to improve the macromolecular orientations.

### 3.5. Morphology of the Fibers

**Figure 9:**
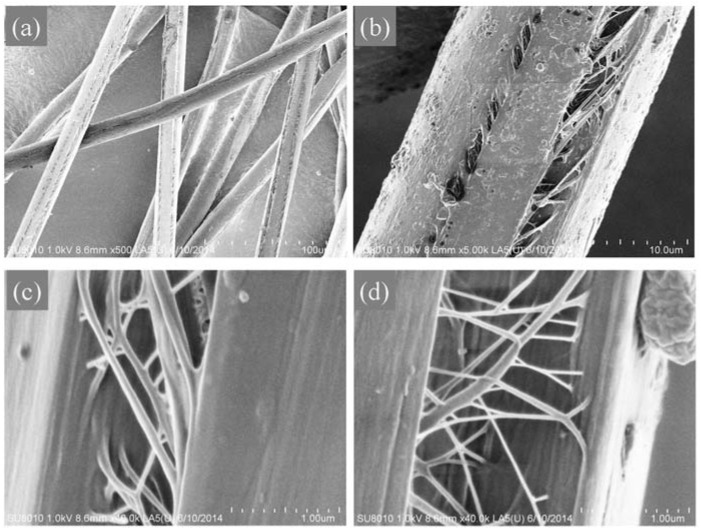
SEM images of fractured surfaces with fibril structures of the PPTA fiber (**a**) and (**b**), and the fibers containing h-PPTA of 3 wt % (**c**) and 8 wt % (**d**).

As we know, PPTA fibers exhibit a skin-core and multi-fibril structures with a large of number of fibrils on the surface that feature a highly orientation along the axial direction of the fiber [19,29,30]. So, the obtained fiber was further etched by 25% dilute sulfuric acid for 15 s to dissolve the amorphous phase on the surface of the fibers. As shown in Figure 9, SEM images display the surficial morphologies of the etched fibers. It is obvious that there exist tearing and separation regions with many fibrils appearing on the surface and inside of the fibers, which could be attributed to the destructive effects of dilute sulfuric acid on the amorphous region with loose structures. There were some crack paths in the fibers, which were alongside the fiber axis, and it was even difficult to distinguish the skin or core parts for the fibers. The crystallinity and orientation level had also an influence on the fibrillation and morphologies of the fibers. From the comparison of the fibrillary morphologies for the PPTA fiber and the blended fibers with h-PPTA in the images of Figure 9, there existed somewhat difference of the fibrillation, and the blended fibers containing h-PPTA gave more splitting fibrils. Crack propagation could be readily occurred parallel to the fiber longitudinal axis because only this requires the interfibrillar rupture [31,32].

## 4. Conclusions

The blended solutions of h-PPTA and the industrial PPTA in concentrated sulfuric acid solvent exhibited a broadened temperature range with the formation of liquid crystalline phase. For instance, the temperature range with the existence of liquid crystalline phenomenon was 78 °C for PPTA/H_2_SO_4_, while the value increased to 117 °C for the solution containing h-PPTA of 8 wt % in the polymer. This extended temperature window could improve the spinnability, which is helpful for the dry-jet wet-spinning technology. The addition of h-PPTA was proved to be favorable to increase the maximum drawing ratio at the air gap in the spinning process and thus enhance the tensile strength and initial modulus of the fibers. Higher molecular weight means a large axial ratio, which is more conducive to the formation of a completely straight rod-like configuration. Moreover, the long-chains of h-PPTA enhanced the interactions of intermolecular and chain segments. The shear stress in the spinneret and the stretching stress at the air gap could induce the orientation of short molecular chains of PPTA along the axial direction of the fibers, and promote the extension of the rod-shaped conformation with the “Oriented Bridging” of h-PPTA long-chains. The crystallinity and orientation level also have a slight influence on the fibrillation of the fibers.

## Figures and Tables

**Table 1:** The crystallinity and macromolecular orientation values of the fibers.

Content of h-PPTA (%)	Crystallinity by XRD (%)	Orientation Factor (*f_c_*) by XRD	Orientation Factor (*f_s_*) by Sonic Velocity
0	55.3	0.804	89.6
3	58.8	0.825	92.0
5	60.1	0.832	93.5
8	61.3	0.859	95.1
10	60.2	0.840	93.9
